# Proteus-Like Syndrome: A Rare Phenotype of Phosphatase and Tensin Homolog Hamartoma Tumor Syndrome

**DOI:** 10.7759/cureus.24135

**Published:** 2022-04-14

**Authors:** Kamil Abu-Shaban, Kenan Bakri, Amber Kihm, Mazzin Elsamaloty, Haitham Elsamaloty

**Affiliations:** 1 Radiology, University of Toledo College of Medicine, Toledo, USA; 2 Radiology, University of Toledo Medical Center, Toledo, USA

**Keywords:** pten hamartoma syndrome, cancer screening, hemihypertrophy, proteus-like syndrome, proteus syndrome, pten

## Abstract

The phosphatase and tensin homolog (PTEN) hamartoma tumor syndrome (PHTS) is a collection of diseases stemming from mutations in the PTEN tumor suppressor gene and is characterized by variable expressivity and abnormal overgrowth in multiple body systems. Its clinical manifestations include, but are not limited to, lipomas, limb overgrowth, dermatologic lesions, and malignancy. The infrequency of occurrence and broadness of clinical presentation has made the diagnosis and differentiation of different subtypes of PHTS challenging. This case report describes a five-year-old patient with a history of autism and macrocephaly who presented to the emergency department with right lower quadrant (RLQ) pain concerning for appendicitis. A physical exam was significant for right leg hemihypertrophy. Imaging ruled out appendicitis but diagnosed two large right-sided abdominal lipomas. The patient was discharged with the recommendation to pursue genetic testing given the physical exam findings and history. Following confirmation of a PTEN tumor suppressor gene mutation, the patient continued to have increased frequency of abdominal pain, developed vision changes, and was diagnosed with a benign follicular thyroid nodule. Hemihypertrophy, recurrent unilateral lipomas, and a confirmed PTEN mutation are consistent with a diagnosis of Proteus-like syndrome, a rare subtype of PHTS.

## Introduction

Phosphatase and tensin homolog (PTEN) is a protein encoded by the PTEN tumor suppressor gene and, along with p53, is one of the most commonly lost tumor suppressor genes [1]. Patients with a germline mutation in the PTEN gene are classified as having PTEN hamartoma tumor syndrome (PHTS) despite the varied clinical features and many subclassified syndromes within PHTS. Previously considered to be separate syndromes, PHTS now includes Cowden syndrome (CS), adult Lhermitte-Duclos disease, Bannayan-Riley-Ruvalcaba syndrome (BRRS), and Proteus syndrome [2,3]. Some of the major clinical criteria of PHTS include macrocephaly, gastrointestinal hamartomas, breast and thyroid cancers, as well as other cancers [2]. We present a case of a patient with a confirmed PTEN mutation whose clinical presentation was consistent with a Proteus-like syndrome with hamartomas confined to the right side of the body. While there have been attempts to clearly delineate the PHTS syndromes, there is significant overlap in clinical presentations between different PHTS syndromes. There has also been a wide variability in clinical presentations between the same PHTS subtypes [2].

## Case presentation

**Figure 1:**
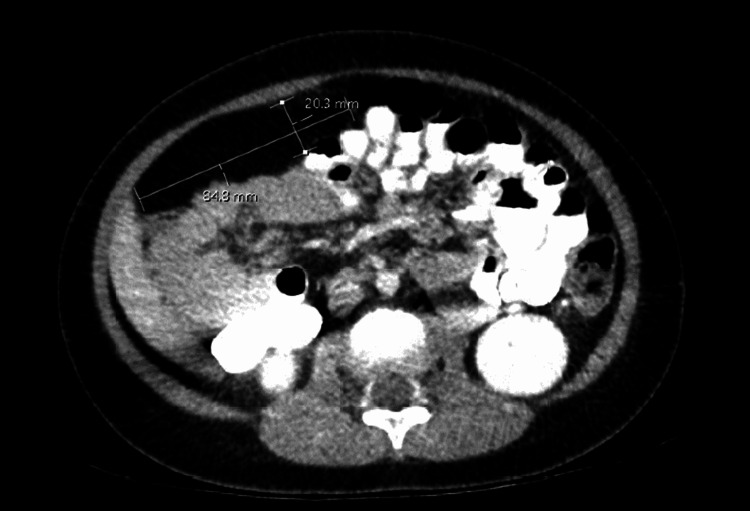
Initial CT abdomen and pelvis – image 1 Focal area of fat in the right upper quadrant adjacent to the inferomedial aspect of the liver measuring 8.5 x 2.0 cm with a mild displacement of the adjacent bowel. This mass raised suspicion of a lipoma CT: computed tomography

**Figure 2:**
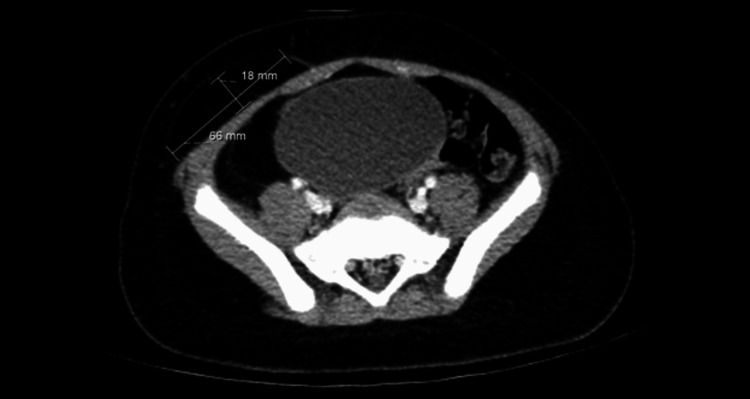
Initial CT abdomen and pelvis – image 2 Focal area of fat in the right lower quadrant anterior to the abdominal wall measuring 6.6 x 1.8 cm consistent with lipoma CT: computed tomography

A five-year-old child presented to the ED with complaints of right lower quadrant (RLQ) pain, nausea, and fever for two days concerning for appendicitis. The patient's past medical history was significant for autism and macrosomia. On physical exam, the patient had right leg hemihypertrophy and macrocephaly. Abdominal ultrasound showed no acute appendicitis. However, two lipomas were found: one in the right upper quadrant (RUQ) and another in the RLQ. CT confirmed an intra-abdominal RUQ lipoma measuring 8.5 x 2.0 cm (Figure 1) as well as a superficial RLQ lipoma measuring 6.6 x 1.8 cm (Figure 2). Given these findings and the patient’s past medical history, they were encouraged to undergo genetic testing. The results confirmed PTEN c. 938Adel frameshift mutation.

**Figure 3:**
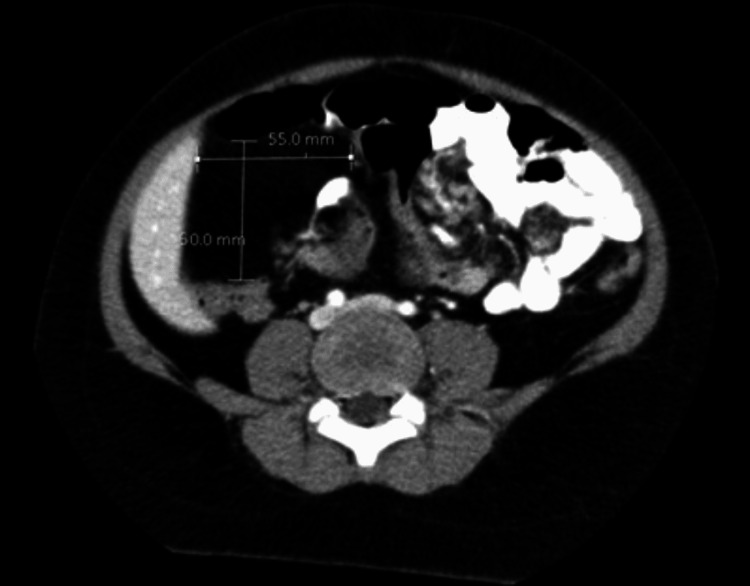
Three-month postoperative CT The image shows expanding focal area of fat in the right upper quadrant that has increased in thickness from a previous study 11 months prior, measuring 5.5 cm x 5.0 cm. There is a moderate displacement of adjacent bowel loops, raising suspicion of a growing lipoma CT: computed tomography

Over the next year, the patient developed worsening abdominal pain, blurry vision, and repeated falls. An MRI of the brain was unremarkable. Roughly eight months after the presentation to the ED, the RLQ lipoma was resected and found to be 12 x 8 x 3 cm on gross examination. Surgical pathology confirmed mature adipose tissue consistent with a lipoma. On a three-month postoperative abdominal and pelvic CT for continuing abdominal pain, the RUQ lipoma had grown in thickness to 5.5 x 5.0 cm with an increased displacement of adjacent loops of the bowel (Figure 3). The patient also had a bone scan for right leg length discrepancy, which revealed a bone age that was two standard deviations beyond the chronological age, according to the standards of Greulich and Pyle.

**Figure 4:**
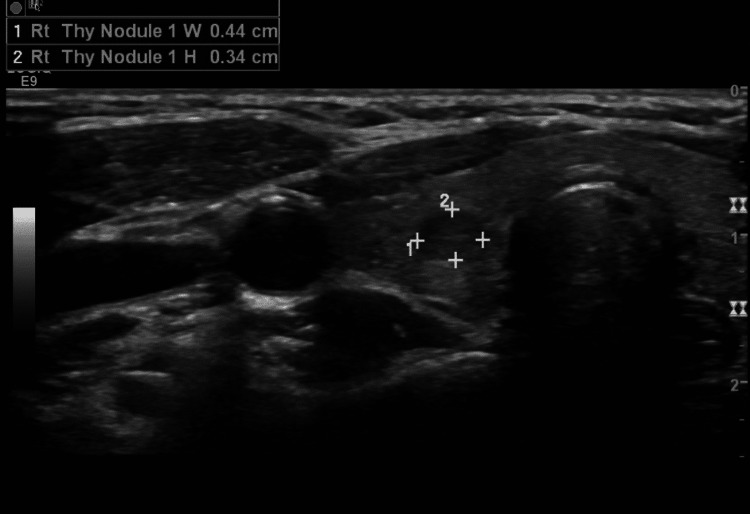
Ultrasound of the patient four years after the initial presentation The image shows a hypoechoic nodule in the right thyroid lobe, measuring 0.4 x 0.3 cm Rt: right. Thy: thyroid. W: width. H: height

**Figure 5:**
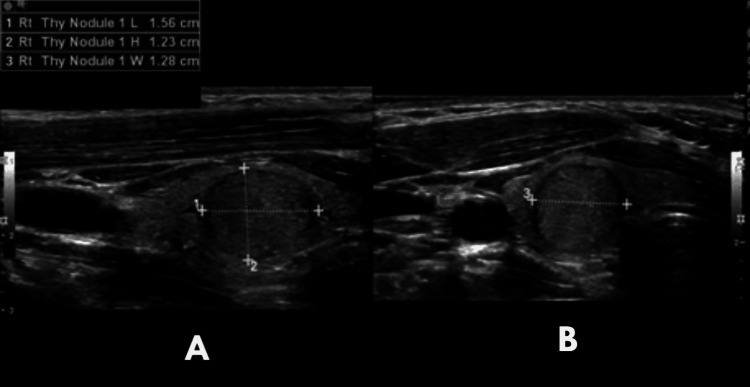
Follow-up ultrasound of the patient The image shows a slightly hypoechoic to isoechoic nodule in the right thyroid lobe seen in a follow-up study two years later. Measurement in the sagittal plane (A) was 1.56 cm x 1.23 cm. The transverse plane (B) width was 1.28 cm Rt: right. Thy: thyroid. L: length. H: height. W: width

Four years after the initial presentation, the patient had a nontoxic right-sided unilateral goiter. An ultrasound found a solid right-thyroid nodule measuring 0.5 x 0.4 x 0.3 cm (Figure 4). Follow-up ultrasound two years later showed an increase in size to 1.6 x 1.2 x 1.3 cm (Figure 5). Due to the increase in size as well as the risk of neoplasia in PTEN syndromes, the ultrasound nodule was biopsied and was found to be benign. General surgery was consulted to discuss the need for the resection of the enlarging nodule.

## Discussion

Proteus syndrome is an extremely rare disease characterized by partial gigantism and hemihypertrophy of the limbs as well as subcutaneous lipomas and verrucous epidermal nevi [4]. As of 2020, there have been only about 200 cases in the literature that delve into the details of Proteus syndrome [5]. Our patient presented with a PTEN mutation and clinical features that resembled Proteus syndrome, as well as unilateral hamartomas on the right side, including abdominal lipomas. Moreover, there have only been 15 cases of Proteus syndrome with abdominal lipomatosis in the literature as of 2009 [6]. The combination of a confirmed PTEN mutation, unilateral hamartomas consistent with PHTS, and hemihypertrophy classified our diagnosis as PHTS with a Proteus-like presentation.

Unlike many PTEN hamartoma syndromes, the diagnostic criteria for Proteus-like syndrome have not been defined [7]. Proteus-like syndrome is diagnosed when individuals exhibit features of Proteus syndrome but do not meet Proteus syndrome diagnostic criteria, which are shown in Table 1 [7]. The patient in the presented case failed to meet the specific category signs for Proteus-like syndrome such as connective and epidermal tissue nevi. However, the patient did meet some characteristics that are part of the diagnostic criteria of Proteus syndrome such as lipomas and disproportionate overgrowth of the limbs. The lack of criteria to define Proteus-like syndrome makes it difficult to adequately diagnose patients correctly, even when a genetic mutation is confirmed. Having a correct diagnosis can enable the patient to undergo the appropriate cancer screening.

**Table 1:** A summary of the diagnostic criteria for Proteus syndrome* *[10]

Diagnostic criteria for Proteus syndrome
General criteria (mandatory)
Mosaic distribution
Progressive course
Sporadic occurrence
Specific criteria (category signs)
Either the single criterion from A or two from B or three from C
Category signs
A
1. Connective tissue nevus
B
1. Epidermal nevus
2. Disproportionate overgrowth (one or more)
Limbs
Skull
External auditory meatus
Vertebrae
Viscera
3. Specific tumors before the end of the second decade
Bilateral ovarian cystadenomas
Parotid monomorphic adenoma
C
1. Dysregulated adipose tissue (either one)
Lipomas
Regional absence of fat
2. Vascular malformation (one or more)
Capillary malformation
Venous malformation
Lymphatic malformation
3. Facial phenotype
Dolichocephaly
Long face
Minor down slanting of palpebral fissures and/or minor ptosis
Low nasal bridge
Wide or anteverted nares
Open mouth at rest

PTEN mutations have been implicated in many different cancers [1]. Therefore, it is not surprising that patients with germline PTEN mutations have an increased risk of developing a wide spectrum of benign and malignant tumors. These include tumors of the brain (i.e., Lhermitte-Duclos disease), breast, endometrial, colon, and thyroid [2]. The lifetime risk for any cancer may be as high as 89%, and the risk is 85% for breast cancer, 35% for thyroid cancer, 28% for endometrial cancer, 34% for renal cancer, 16% for colorectal cancer, and 5% for melanoma. This is in addition to the high prevalence of benign tumors of the skin (i.e., trichilemmomas), thyroid (i.e., adenomas), and GI tract (i.e., polyps) [7]. Current guidelines for cancer screening in PHTS patients, such as those published by the National Comprehensive Cancer Network, are quite complex given the multiple cancer risk. Current recommendations include an annual comprehensive physical exam starting at age 18, annual mammography with MRI starting at age 25, and an annual thyroid ultrasound starting at age seven. Additional screening for renal and endometrial cancers is also recommended [8,9].

## Conclusions

Proteus-like syndrome is a subtype of PHTS; it has been poorly described in the literature due to a lack of diagnostic criteria and variable phenotype. This case report described a presentation of PHTS with lower limb hemihypertrophy, a Proteus syndrome characteristic, accompanied by other growth abnormalities, including unilateral recurrent lipomas, a follicular thyroid nodule, and macrocephaly. PHTS patients are at an increased risk of various cancers. Earlier diagnosis will lead to earlier screening and the potential for increased longevity. More data on PHTS patients is needed to optimize benefit versus harm when it comes to screening for these patients.
